# A reproductive history of mothers with spina bifida offspring-a new look at old issues

**DOI:** 10.1186/1743-8454-3-10

**Published:** 2006-08-01

**Authors:** Thomas L Farley

**Affiliations:** 1 Arkansas Spinal Cord Commission, 1501 North University Avenue, Little Rock, Arkansas, 72207-5233, USA

## Abstract

**Background:**

Spina bifida is a disorder of the cerebrospinal fluid system associated with failure of neural tube closure in the fetus. Reproductive history studies of mothers with spina bifida offspring have often been conducted shortly after the affected child's birth. In this study, a large group of community-based mothers were studied after most had completed their families. The aims were to present a more comprehensive reproductive history and to test several hypotheses regarding the nature of spina bifida.

**Methods:**

Data from 271 mothers was collected by interview 18.3 mean years after the affected child's birth. Data analysis was by χ-square, Fisher exact test and t test with a *p *value less than 0.05 considered significant.

**Results:**

Females made up 56.5% of affected offspring (probands) and 53.1% of unaffected offspring. The spina bifida and anencephaly recurrence rate was 4.0%. The twinning rate was 8.6/1000 live births. 24.4% of mothers had a history of spontaneous abortion and the rate varied by pregnancy order from 87 to 185/1000 live births. Duration of pregnancies subsequent to probands was shorter for female than male probands. Mean birth weight of probands with high lesions exceeded those with low lesions. A spontaneous abortion preceded female probands more often than males as compared to live births. Affected males with high lesions conceived by white mothers were at greater risk to be spontaneously aborted. Previous inter-gestational interval for mothers with no history of spontaneous abortion was longer for probands than unaffected offspring but not for mothers with a history of spontaneous abortion.

**Conclusion:**

Overall, and for every major subgroup of these mothers, more affected and unaffected female than male offspring were born. Differences by gender and lesion level among probands and between probands and unaffected offspring were consistent with an etiology of unknown genetic factors, hormonal and/or immune system factors.

## Background

Spina bifida is a disorder of the cerebrospinal fluid system resulting from a failure of neural tube closure in the fetus and associated with accompanying deformities leading to hydrocephalus. Over the past twenty-five years, a number of studies [1] have demonstrated that environmental and genetic factors play an important part in neural tube defect (NTD) etiology. Dietary folic acid fortification [2] and supplementation [3], in particular, have shown success as preventative measures. However, despite such progress, much is still unknown about the complex, multi-factorial etiology of NTD.

This study had two purposes. The first was to describe and analyze variables related to the reproductive history of mothers who had given birth to a child with spina bifida (affected). This was carried out by investigating a large group of mothers who had given birth to spina bifida offspring residing in the state of Arkansas in the United States. The second purpose was to use the findings to test four hypotheses regarding the nature of spina bifida. These hypotheses were: 1) the occurrence of twins is related to the incidence of spina bifida [4,5]; 2) a preceding spontaneous abortion is associated with the presence of spina bifida in the following pregnancy [6]; 3) spina bifida offspring are associated with tissue remaining from a previously aborted conceptus [7,8] ; 4) more males than females affected with spina bifida are lost prenatally resulting in a preponderance of affected females at birth [9,10].

No outside control group was used for this descriptive study. However, within the described population, one subgroup was compared to another subgroup for one or more variables in order to clarify a relationship or to test a hypothesis.

## Methods

The Arkansas Spinal Cord Commission (ASCC), a state agency, is mandated by state law to maintain a registry of all residents with spinal cord disability including individuals with spina bifida. Subjects for this study were drawn retrospectively from the ASCC registry and consisted of 271 mothers with information about their offspring. Information about the child affected with spina bifida (proband) was collected in 1993; reproductive history and other information about the child's biological mother were collected in 1995–1996.

The 1993 study surveyed all known Arkansas residents with spina bifida. The purpose was to determine the type and prevalence of secondary and other associated conditions in order to provide appropriate services. ASCC Case Managers interviewed individuals/families in their home with the child's mother present when possible to assist in answering questions. The survey instrument was designed by a panel of health care professionals and consumers experienced in working with individuals with spina bifida. Lesion level was determined by muscle action against gravity. Thoracic level lesions were defined as high lesions and lumbar and sacral level lesions defined as low lesions using an established definition [11]. A shunt was counted as present if ever installed during the child's lifetime. Shunts are installed to drain excess cerebrospinal fluid from the ventricles and reduce pressure within the head and are an indicator of hydrocephalus. Out of a possible 419 individuals available for contact, 380 were interviewed for a completion rate of 91%. The remainder refused to participate, could not be located or had died during the study time period. This study [12] has been previously reported.

The 1995–1996 study surveyed the biological mothers of individuals with spina bifida who were questioned in 1993. The purpose was to determine the reproductive history and a selective medical and behavioral history. Mothers were asked to respond to a mailed questionnaire developed by ASCC staff. Mothers who did not respond by mail were contacted by telephone for questionnaire completion. Twins were counted as one pregnancy and one birth if both fetuses survived. For affected/unaffected twins, the gender and affected status of the affected child was used to designate the gender and affected status of the pregnancy. For affected/affected twins, the gender of the first delivered twin was designated the gender of the pregnancy. Spontaneous abortions were defined as non-induced delivery of the fetus at 28 weeks or less after conception. Twenty-eight weeks was chosen because it was a natural break in the distribution of the data and for comparison with prior research [13]. Stillbirths were defined as delivery of a non-live fetus at 29 weeks or more. Stillbirths and spontaneous abortions were counted for the purpose of determining pregnancy order. Stillbirths were not counted as spontaneous abortions. Cases with stillbirths were excluded from analysis when comparing mothers with a history of spontaneous abortion (MWH) and mothers with no history of spontaneous abortion (MWNH) in order to avoid how stillbirths should be classified. The inter-gestational interval (IGI) between pregnancies was measured in days using the dates the pregnancies were terminated by a live birth, spontaneous abortion or stillbirth. Paternity was determined for all offspring. Natural fathers were defined as the biological fathers of the proband. Partners were defined as all the fathers of the mother's children. All responses were by self-report.

Of the 380 mothers who participated in the 1993 study, 282 completed the questionnaire for a completion rate of 74.2%. The primary reasons for non-completion were the biological mother could not be located (71), or was deceased (6); followed by no information known about the mother (7) or the child was adopted (5) with no further contact with the biological mother. Other reasons for non-completion included death of the child (5) during the 1995–1996 study time period and refusals (4). Also, nine mothers with induced abortions were excluded for a total of 271 mothers and offspring information. Proxies, answering for the mother, completed 20 of the 271 maternal questionnaires. These proxies were knowledgeable about the mother's pregnancy history and thus, not excluded from the analysis.

Statistical Package for the Social Sciences was used for statistical analysis. Tests for significance used χ-square and Fisher's exact test for contingency tables and student's T test for two independent samples with rejection of the null hypothesis at the 0.05 level. Both the 1993 and the 1995–1996 studies were approved by the University of Arkansas for Medical Sciences Human Subjects Review Committee.

## Results and discussion

Overall, the 271 subjects were 71.3% of those families completing the 1996 survey and 64.7% of the 419 families with a spina bifida (SB) child in Arkansas in 1993. The group was geographically diverse with subjects residing in 64 of Arkansas' 75 counties.

### Demographics (Table 1)

**Table 1:** Demographic and related characteristics of mothers and probands

**Race of mother**	Number	Percent
Caucasian	237	87.5
African-American	33	12.2
Asian/Pacific Islander	1	0.4
		
**Age of mother**	Mean years	
At proband's birth	25.2	
At time of survey	43.5	
		
**Pregnancy outcome**	Number ^(1)^	Percent
Live birth	701	87.7
Spontaneous abortion	95	11.9
Stillbirth	3	.4
		
**History of spontaneous abortion**	Number	Percent
No	205	75.6
Yes	66	24.4
		
**Pregnancy distribution**	Range	Mean
All pregnancies	1 to 12	2.95
Live births	1 to 12	2.59
Spontaneous abortions	1 to 6	1.42
		
**Partners of mothers**	Number	Percent
One partner	211	77.9
Two partners	48	17.7
Three partners	9	3.3
Four partners	3	1.1
		
**Offspring by partner of mother**	Number	Percent
271 natural fathers of proband	697	87.2
60 fathers of proband's half-siblings	102	12.8
		
**Proband gender**	Number	Percent
Female	153	56.5
Male	118	43.5
		
**Proband lesion level**	Number	Percent
Thoracic	110	40.6
Lumbar	135	49.8
Sacral	21	7.7
Missing	5	1.8

Note: ^(1) ^One mother had a history of spontaneous abortion and also had a stillborn child.

Most of the mothers were Caucasian (87.5%) and produced children with only one partner (77.9%). A history of spontaneous abortion was present in 24.4% of the mothers. On average, mothers were interviewed 18.3 years after the birth of the affected child. Most of the 799 pregnancies were live births (87.7%). The wide range in the number of pregnancies and live births was due to a few data outliers. Natural fathers of the proband were responsible for 87.2% of all live births. The lesion level of 40.6% of all probands was classified as thoracic or a high lesion level. Live-born affected females exceeded males by 56.5 to 43.5%.

### Multiple births

Seven sets of twins were produced. One set consisted of 2 affected females, three sets of affected female and unaffected female, one set of unaffected females (conjoined, died second day), one set of unaffected males (died one hour after birth), and one set of unknown affected status and gender (spontaneously aborted at 14 weeks). No other multiple births were reported.

The high percentage of females among twins is consistent with a previous report [14].

**Hypothesis 1**: the occurrence of twins is related to the incidence of spina bifida.

Twins were a small subset of total pregnancies-only seven sets of twins occurred in 701 live births and in 799 pregnancies. The twinning rate was 8.6 compared to 25.9 per 1000 live births for the United States in 1996 [15]. Affected offspring were found among twins but no evidence was found to support the hypothesis that twins are causally related to the development of spina bifida or that the interaction of twin embryos [4] results in a loss of male probands. In fact, this group of mothers produced twins at a lower rate than the general population.

### NTD recurrence risk and other associative conditions

No mother, natural father or other partner was affected with a NTD. There were thirteen (10 spina bifida and 3 anencephaly) recurrences of NTD reported by eleven mothers. Two mothers reported two recurrences of SB. No mother reported an NTD recurrence with different partners. Regarding other disability conditions, 36 mothers reported 40 children with a variety of problems: mental retardation (7), asthma (5), orthopaedic problem (4), slow learner (3), eye problem (3), congenital heart disease (2), ear problem (2), back bone abnormality (2), seizures (1), diabetes (1), hypospadias (1), conjoined twins (1), dyslexia (1), Bells Palsy (1) and abnormality of lungs (1), lip-cleft (1), bowel (1), intestine (1), kidney/uterus (1), ureter/kidney (1). No mother reporting a second child with NTD reported any other disability condition.

The rate of NTD (spina bifida and anencephaly) recurrence was 4.0% and slightly higher than the 3.0% to 3.2% previously reported rates in the United States [16-18] and 2.2% in Alberta, Canada [19].

In addition to NTDs, other disabling conditions in the offspring of these mothers have been previously reported in probands [17,20,21] and siblings [17,21]. The wide variety of physical abnormalities, mental retardation and other conditions suggests these mothers were not only at high risk for producing offspring with NTDs but for other conditions and disabilities as well.

### Pregnancy duration

The pregnancy duration of the 701 live births ranged from 22 to 43 weeks with a mean of 38.6 weeks. The 95 spontaneous abortions ranged from 3 to 28 weeks with a mean of 10.7 weeks. The duration of the three stillbirths was 36, 36 and 40 weeks with a mean of 37.3 weeks. The pregnancy duration of all probands ranged from 26 to 43 weeks with a mean of 39.5 weeks for both males and females.

The duration of pregnancies immediately subsequent **to **probands differed by proband gender (Table 2). For non-first and non-last pregnancy probands of all mothers, the mean duration of the subsequent pregnancy was significantly shorter for females than males (*p = *0.029).

**Table 2:** Duration (weeks) of pregnancies previous to and subsequent from proband by gender, history of spontaneous abortion

	Male proband		Female proband		
	N	Mean	N	Mean	*p *value
**All mothers**					
Previous pregnancy	69	36.9	100	33.5	n.s.
Subsequent pregnancy	46	38.6	58	34.6	0.029
					
**Mothers with history**					
Previous pregnancy	14	27.9	33	20.9	n.s.
Subsequent pregnancy	8	32.8	19	24.5	n.s.
					
**Mothers with no history**					
Previous pregnancy	55	39.2	67	39.6	n.s.
Subsequent pregnancy	38	39.9	39	39.6	n.s.

The same data pattern was present for MWH although statistically not significant. For MWNH, the duration of both previous and subsequent pregnancies were essentially equal. Thus, the difference in pregnancy duration for all mothers was due to the difference in pregnancy duration for mothers with a history of spontaneous abortion.

### Outcome of previous and subsequent pregnancies

For all mothers, the outcome of pregnancies immediately previous to the proband differed by proband gender (Table 3). More spontaneous abortions occurred previous to female than male probands (*p = *0.031).

**Table 3:** Number of previous and subsequent pregnancies to proband by pregnancy outcome, all mothers

	Proband		
	Male	Female	*p *value
**Previous pregnancy**			
Live birth	67	68	0.031
Spontaneous abortion	9	24	
**Subsequent pregnancy**			
Live birth	44	50	n.s.
Spontaneous abortion	5	12	

Abortuses previous to [6,13,17,22-25] and subsequent to [13] NTD offspring have been documented. Although previous abortuses have been suggested to increase the risk of NTD embryogenesis [6,22-25], abortuses subsequent to probands suggest that abortus proximity to probands is not a causative but a temporal [26] relationship. The difference in the outcome of pregnancies previous to probands depending on proband gender is consistent with an interaction of unknown maternal-foetal hormones or an immune system response.

### Inter-gestational intervals

To determine if the immediately previous and subsequent IGI differed between probands and unaffected offspring, only MWNH were analyzed to eliminate any effect of spontaneous abortions. The IGI between pregnancy 1 and pregnancy 2 was analyzed (Table 4). For all MWNH the IGI previous to unaffected second-born offspring was shorter than the IGI previous to second-born probands (*p = *0.014); however, the IGI subsequent to first-born unaffected offspring was not statistically different from the IGI subsequent to first-born probands. The IGI previous to second-born unaffected male and female offspring were shorter than the IGIs previous to second-born male and female probands but the result was not statistically significant. For the same groups, there was less difference between subsequent IGIs that were not statistically significant.

**Table 4:** Previous ^(1) ^and subsequent ^(2) ^inter-gestational interval (days) between pregnancy 1–2 for MWNH ^(3) ^by proband gender.

	Probands		Unaffected		
	N	Mean	N	Mean	*p *value
**All MWNH**					
Previous	74	1375.8	94	1070.3	0.014
Subsequent	49	1180.1	122	1208.5	n.s.
					
**MWNH, male offspring**					
Previous	29	1425.9	46	1134.5	n.s.
Subsequent	28	1193.8	55	1126.3	n.s.
					
**MWNH, female offspring**					
Previous	45	1343.5	48	1008.7	n.s.
Subsequent	21	1161.8	67	1275.9	n.s.

Notes: ^(1) ^Previous-calculated from second-born probands. ^(2) ^Subsequent- calculated from first-born probands. ^(3) ^MWNH = mothers with no history of spontaneous abortion. Cases with stillbirths were excluded from the analysis.

Also analyzed was the immediately previous and subsequent IGI for all mothers, for MWNH and for MWH; however, no significant differences were found by proband gender (results not shown).

Longer previous intervals for probands were found as compared to unaffected offspring as reported by another [13]; however, shorter previous intervals have also been found [23]. The difference between subsequent intervals for probands and unaffected offspring was not statistically significant as previously reported [13]. The inclusion of stillbirths in the analysis of Table 4 would have resulted in a statistically significant difference (*p *= 0.007) in previous IGI between male probands and unaffected offspring.

### Characteristics of previous inter-gestational intervals

To further delineate the characteristics of inter-gestational intervals previous to probands, IGI was analyzed between probands and unaffected offspring by a history of spontaneous abortion and by proband lesion level (Table 5). For all mothers, previous IGI was shorter for unaffected offspring than for probands (*p *= 0.004). Also for MWNH, previous IGI was shorter for unaffected offspring than for probands (*p *= 0.014). However, there was no significant difference in previous IGI between unaffected offspring and probands for MWH. In addition, no significant difference in previous IGI was found between unaffected offspring and probands for MWH where the mother's first pregnancy was a spontaneous abortion. For MWNH, previous IGI was shorter for unaffected offspring than for probands with a high lesion (*p *= 0.037); however, the same analysis found no significant difference for low lesion probands.

**Table 5:** Previous ^(1) ^inter-gestational interval (days) for pregnancy order two probands and unaffected offspring by maternal and child characteristics.

	Probands		Unaffected		
	N	Mean	N	Mean	*p *value
All mothers	89	1272.7	141	974.7	0.004
All MWH ^(2)^	15	764.2	47	783.3	n.s.
All MWNH ^(3)^	74	1375.8	94	1070.3	0.014
MWH, PG1 was spontaneous abortion	10	767.3	17	632.9	n.s.
MWNH and high lesion proband	29	1620.6	41	1135.9	0.037
MWNH and low lesion proband	44	1167.9	52	1022.7	n.s.

Notes: ^(1) ^Previous is calculated for pregnancy order two offspring. ^(2) ^MWH = mothers with history of spontaneous abortion. ^(3) ^MWNH = mothers with no history of spontaneous abortion. Cases with stillbirths were excluded from all groups in the analysis.

**Hypothesis 2**: a preceding spontaneous abortion is associated with the presence of spina bifida in the following pregnancy.

Several findings fail to support this hypothesis: First, only 24.4% of the mothers had a history of spontaneous abortion; thus, mothers with no history of spontaneous abortion produced the majority of affected offspring. Second, as previously stated, abortuses subsequent from probands suggest that abortus proximity to probands is not a causative but a temporal relationship. Third, for all mothers, previous IGI was significantly longer for probands than unaffected offspring; however, this was only true for MWNH not for MWH. Thus, IGI length was not related to recognized spontaneous abortion but to unknown factor(s) associated with MWNH. Also, the length of the previous IGI was not significantly different by affected status for MWH when the first pregnancy is a spontaneous abortion.

**Hypothesis 3**: spina bifida offspring are associated with tissue remaining from a previously aborted conceptus.

There are two explanations regarding spontaneous abortion and longer IGIs preceding affected rather than unaffected offspring. These hypotheses have previously been explored and well summarized [6]. Briefly, the first states that tissue, a trophoblastic cell rest (TCR), remaining from the previous spontaneous abortion interferes with the normal embryogenesis of the following pregnancy resulting in an affected offspring/product. Longer IGIs for affected offspring are the result of TCR aborting unrecognized pregnancies before the next recognized pregnancy. The second states that both the previous spontaneously aborted fetus and the following pregnancy were affected.

The findings of this paper have found no support for trophoblastic cell rest, as measured by IGI, as a cause for spinal bifida. Although longer IGIs were found previous to unaffected offspring than for probands, the difference was significant for MWNH and not for MWH as predicted by the TCR hypothesis. Instead, the findings suggest that previously aborted as well as subsequently aborted fetuses are affected pregnancies.

The lack of a relationship between IGI and affected status for MWH plus the presence of such a relationship for MWNH, suggests that the pathogenesis for affected offspring may be different for MWH and MWNH. It may be that the same unknown factor(s) are present for both MWH and MWNH but the presence of spontaneous abortions hides its detection for MWH. The finding that IGI and affected status was related for MWNH with male probands and probands with high lesions, suggests a genetic aetiology.

### Birth weight

The birth weight of probands differed by high and low neurological lesion level. The mean birth weight of 107 probands with high lesions was 114.0 ounces and 108.5 ounces for 147 probands with low lesions (*p = *0.041).

The heavier birth weight of probands with high lesions suggests a genetic factor. Since probands with high lesions are also significantly related to IGI, these unknown genetic factor(s) may be the same.

### Spontaneous abortion

The spontaneous abortion rate varied by pregnancy order (Table 6) and each pregnancy order for the study group was significantly higher than the state rate (*p *< 0.0001 for each pregnancy).

**Table 6:** Spontaneous abortion rate for Arkansas and study population

	Live Births	Spontaneous Abortions	Rate per 1000 Live Births	*p *value ^(1)^
State of Arkansas ^(2)^	36,356	930	25.58	----
				
All mothers ^(3)^				
Pregnancy 1	240	31	129.16	< 0.0001
Pregnancy 2	218	19	87.15	< 0.0001
Pregnancy 3	124	23	185.48	< 0.0001
Pregnancy 4	71	11	154.90	< 0.0001

Notes: ^(1)^X-square p values calculated using data for each pregnancy and state of Arkansas data. ^(2) ^Data are for year 1996; spontaneous abortions defined as < 20 weeks. ^(3) ^For comparison purposes, study population rates calculated as if all pregnancy outcomes for a particular pregnancy order occurred in one year. Cell frequencies for pregnancy 5 and greater were too small for analysis.

Although the spontaneous abortion rate is 3.4 to 7.3 times higher than the 1966 general Arkansas population rate of 25.58 per 1000 live births [27], only 24.4% of the mothers had a history of spontaneous abortion. Thus, most mothers did not have a history of spontaneous abortion.

### Selected maternal characteristics by history of spontaneous abortion

There were no significant differences between mothers with and without a history of spontaneous abortion in terms of race, age at proband's birth and age at interview (Table 7). Although MWH had more pregnancies than MWNH (*p <*0.001), both groups had, on average, about the same number of live births.

**Table 7:** Selected characteristics of mothers by history of spontaneous abortion

Characteristic	Measure	MWNH ^(1) ^N = 202	MWH ^(2) ^N = 66	*p *value
White race (Caucasian)	Percent	86.1	90.9	n.s. ^(3)^
Number of pregnancies	Mean	2.61	3.94	< 0.001
Number of live births	Mean	2.61	2.52	n.s.
Age at proband's birth	Mean years	25.27	24.91	n.s.
Age at interview	Mean years	44.22	41.31	n.s.

Notes: ^(1) ^MWNH = mothers with no history of spontaneous abortion. ^(2) ^MWH = mothers with history of spontaneous abortion. Cases with stillbirths excluded from the analysis. ^(3) ^Fisher exact *p *value calculated on cell frequencies (not shown).

The greater number of pregnancies by MWH than MWNH indicates that MWH changed their fertility patterns in response to spontaneous abortion. This compensation resulted in mothers with and without a history of spontaneous abortion to give birth, on average, to the same number of live-born children.

### Preponderance of female offspring

Females made up 56.5% of affected offspring and 53.1% of unaffected offspring. Overall, and for both MWH and MWNH groups, there was a majority of female offspring; however, female preponderance was most pronounced among affected offspring (Table 8). For all mothers proband gender was significantly different for MWH and MWNH (*p = *0.045) but the gender of unaffected offspring were not.

**Table 8:** Offspring gender by affected status for maternal history of spontaneous abortion, number of partners

	MWH ^(1)^		MWNH ^(2)^		
	Male	Female	Male	Female	*p *value
**All mothers**					
Probands	21	45	94	108	0.045
Unaffected	45	54	158	168	n.s.
					
	MWH		MWNH		
	Male	Female	Male	Female	*p *value
**Mothers with one partner**					
Probands	13	35	76	85	0.013
Unaffected	32	33	114	124	n.s.
					
	MWH		MWNH		
	Male	Female	Male	Female	*p *value
**Mothers with 2+ partners**					
Probands	8	10	18	23	n.s.
Unaffected	13	21	44	44	n.s.

Notes: ^(1) ^MWH = mothers with history of spontaneous abortion. ^(2) ^MWNH = mothers with no history of spontaneous abortion. Cases with stillbirths were excluded from the analysis.

**Hypothesis 4**: more males than females affected with spina bifida are lost prenatally resulting in a preponderance of affected females at birth.

The significant association between a history of spontaneous abortion and gender for affected offspring and not for unaffected offspring supports the conclusion that spina bifida is responsible for the decrease of affected males in comparison to affected females. In addition, the greater number of spontaneous abortions before and after female probands (Table 3) suggests these spontaneous abortions were most likely males and affected with spina bifida. Thus, the difference in gender for affected offspring is consistent with the prenatal loss of males. These data suggest that spina bifida does not occur more frequently among females but that spina bifida occurs more frequently among "surviving," live-born, females.

The lack of a significant association between a history of spontaneous abortion and gender for unaffected offspring suggests factor(s) other than spontaneous abortion to be responsible for the preponderance of females among unaffected offspring.

### Multiple partners

For mothers with one partner, proband gender was significantly different for MWH and MWNH (*p = *0.013) but the gender of unaffected offspring were not. For mothers with two or more partners, proband gender was not significantly different for MWH and MWNH and neither was the gender of unaffected offspring.

For MWH and one partner, the gender of probands was significantly different than unaffected offspring (*p = *0.021) and also for the expected gender outcome (*p = *0.035, Table 9). The analysis was restricted to mothers with one partner to eliminate the genetic influence of other partners.

**Table 9:** Proband gender outcome for mothers with a history of spontaneous abortion and one partner by unaffected offspring and expected gender outcome

Probands		Unaffected offspring		*p *value
Male	Female	Male	Female	
13	35	32	33	0.021
				
Probands		Expected outcome		*p *value
Male	Female	Male	Female	
13	35	24	24	0.035

The significance difference in proband gender by a history of spontaneous abortion for mothers with one partner and not for mothers with two or more partners (Table 8) suggests a genetic aetiology. These unknown factor(s) not only appear to influence the presence of spina bifida but gender as well.

For MWH and one partner, the significant difference in gender between probands and unaffected offspring and also expected gender outcome suggests that genetic factor(s) play an important role in the spontaneous abortions of this group of mothers.

### Race, lesion level and presence of shunt

Mother's race, proband lesion level, and presence of a shunt were not significantly associated with proband gender (results not shown). Proband gender, however, was significantly associated with a history of spontaneous abortion for white mothers (*p = *0.049), probands with a high lesion level (*p = *0.027) and probands with a shunt installed (*p = *0.020, Table 10).

**Table 10:** Proband gender by mother's history of spontaneous abortion for selected characteristics

	MWNH ^(1)^		MWH ^(2)^		
	Male	Female	Male	Female	*p *value
**Mother's race (3)**					
White	82	92	19	41	0.049
NonWhite	13	15	2	4	n.s.
					
**Proband Lesion Level**					
High	40	40	7	21	0.027
Low	54	64	13	24	n.s.
					
**Proband Shunt installed?**					
Yes	70	77	14	36	0.020
No	24	29	7	9	n.s.

Notes: ^(1) ^MWNH = mothers with no history of spontaneous abortion. ^(2) ^MWH = mothers with history of spontaneous abortion. Cases with stillbirths were excluded from the analysis. ^(3) ^Whites are Caucasian; non-whites are African-American and Asian/Pacific Islander.

The greater preponderance of affected females found for MWH indicate the characteristics of most of the affected males which have been lost prenatally: those of white mothers, with a high lesion level and those which would have required a shunt had they lived.

### Longer retrospective view

This study interviewed mothers an average of 18.3 years after the proband's birth, enabling information to be collected on the mother's completed reproductive history including: pregnancies and offspring after the birth of the affected child, disabilities in siblings and multiple partners. Thus, a more complete picture of the mother's reproductive history was obtained through a longer retrospective view.

Historically, much of the spina bifida literature on sex ratios is descriptive studies of probands. Because many of these studies were triggered by the birth of an affected child, the analysis focused on affected individuals, the family and the environment immediately before and after the birth of the proband. Although control groups or larger reference groups may be used for comparison, the results may look quite different when, years later, the mother's total reproductive history is known.

### Study limitations

Cases with multiple births (twins) and cases of mothers with more than one partner were included in order to describe the entire reproductive history. A genetic study of the same group may want to limit cases to singleton births and exclude partners of the mother that are not the proband's natural father. Including these variables in the present analysis allowed the discovery of new findings.

The exclusion of three cases with stillbirths in the comparison of MWH and MWNH avoided controversy as to how stillbirths should be classified. However, it also excluded an important trait of the reproductive history of these mothers and reduced the number of cases available for analysis.

The amount of time after the birth of the proband before the mother was interviewed may be seen as excessive. When investigating extra-somatic environmental variables such as toxin exposure or food intake, a much shorter time lapse is desirable in order to reduce memory recall bias. However, in this study, recall bias was not a problem-there were no mothers who could not report the gender, birth date and father of all their children.

Determination of lesion level by sensation performed by a trained physician in a clinical setting would have been preferable to observing muscle action against gravity conducted in the subject's home.

The subjects of the present study are mothers who had children with spina bifida living during the previously stated time periods. Mothers who had a child with spina bifida that later died before the study time periods were not included. Although identifying and locating these mothers would have been difficult if not impossible, their inclusion would offer a more comprehensive view of the reproductive history of all mothers to have given birth to spina bifida offspring.

## Conclusion

The reproductive history of this high-risk NTD group of mothers indicates many abnormal characteristics. In addition to the presence of NTD offspring, the preponderance of females among affected and unaffected offspring, a high rate of spontaneous abortion, the presence of non-NTD disabilities and a lower than expected production of twins set this group of mothers apart from the general population. Within this group of mothers, subgroup differences in pregnancy duration, a history of spontaneous abortion, inter-gestational intervals, proband gender, the number of partners and proband neurological lesion level indicate a heterogeneous multi-factorial etiology. Unknown genetic factors, hormonal and/or immune system interactions are hypothesized to be associated with spina bifida and offspring gender.

## Competing interests

The author(s) declares that he has no competing interests.

## Authors' contributions

Sole Author. The author has read and approved the final version of this paper
